# Electrospun Phospholipid Fibers as Micro-Encapsulation and Antioxidant Matrices

**DOI:** 10.3390/molecules22101708

**Published:** 2017-10-17

**Authors:** Elhamalsadat Shekarforoush, Ana C. Mendes, Vanessa Baj, Sophie R. Beeren, Ioannis S. Chronakis

**Affiliations:** 1 Nano-Bio Science Research Group, DTU-Food, Technical University of Denmark, Kemitorvet 202, 2800 Kongens Lyngby, Denmark; elsh@food.dtu.dk (E.S.); ioach@food.dtu.dk (I.S.C.); 2 DTU-Chemistry, Technical University of Denmark, Kemitorvet 207, 2800 Kongens Lyngby, Denmark; vanbaj@kemi.dtu.dk (V.B.); sopbee@kemi.dtu.dk (S.R.B.)

**Keywords:** phospholipids, electrospinning, microfibers, antioxidants, encapsulation, vanillin, curcumin

## Abstract

Electrospun phospholipid (asolectin) microfibers were investigated as antioxidants and encapsulation matrices for curcumin and vanillin. These phospholipid microfibers exhibited antioxidant properties which increased after the encapsulation of both curcumin and vanillin. The total antioxidant capacity (TAC) and the total phenolic content (TPC) of curcumin/phospholipid and vanillin/phospholipid microfibers remained stable over time at different temperatures (refrigerated, ambient) and pressures (vacuum, ambient). ^1^H-NMR confirmed the chemical stability of both encapsulated curcumin and vanillin within phospholipid fibers. Release studies in aqueous media revealed that the phenolic bioactives were released mainly due to swelling of the phospholipid fiber matrix over time. The above studies confirm the efficacy of electrospun phospholipid microfibers as encapsulation and antioxidant systems.

## 1. Introduction

Phospholipids have been used for preparing biomimetic capsular structures (mainly vesicles or liposomes) [1,2,3,4], for several life science applications, including nano-micro encapsulation of drugs [5] and mammalian cells [6], and in food [7] as delivery carriers of nutrients, nutraceuticals, food additives and antimicrobials. Encapsulation of bioactives within lipid formulations often offers enhanced stability and protection, combined with superior biocompatibility and enhanced permeability, depending on the lipid composition and properties [8,9]. Among other phospholipids, asolectin, constituted by a mixture of lecithin, cephalin and phosphatidylinositol, saturated fatty acids, mono-unsaturated and poly-unsaturated fatty acids has been used to develop nano-microstructures such as fibers [10,11,12,13], hydrogels [14] and liposomes [7,15] for the encapsulation of bioactives [13,16]. In addition, asolectin components have also been proven to display antioxidant properties [16,17,18,19]. Pan and co-workers [16] evaluated the effect of the antioxidant properties of lecithin emulsifier on the oxidative stability of encapsulated bioactive compounds. They demonstrated that the antioxidant activity of lecithin emulsifier can significantly reduce the saturation of free radicals across the interface of oil-in-water emulsions, as well as the rate of oxidation of the bioactive encapsulate (curcumin), thus increasing its shelf life. The antioxidant effect of lecithins was also tested on several oils and fats varying in FA composition and tocopherol content [17]. They found that lecithins, at specific concentrations, exhibited a good protective effect against oxidation on several oils and fats with varying FA composition. These antioxidant properties were enhanced in samples containing tocopherols, due to the synergistic interactions between amino-alcohol phospholipids and γ- and δ-tocopherols. Furthermore, Doert and co-authors [20] studied the synergistic effect of lecithins with tocopherols and observed that phospholipids synergistically enhance the antioxidant effect of phenolic antioxidants.

Phenolic compounds are known for the inhibition of free radical formation and/or for the interruption of the propagation of autoxidation [21]. Vanillin (4-hydroxy-3-methoxybenzaldehyde) is a phenolic compound, which can either be extracted from pods of *Vanilla Planifolia* or synthesized chemically, and has been widely used in the food industry as a flavor, but also as a food preservative, due to its antioxidant, antimicrobial, anticarcinogenic and antimutagenic properties [22,23]. However, its high volatility and thermal instability are the main drawbacks for its use and processing. Curcumin is another phenolic compound derived from the turmeric of the herb *Curcuma longa* L., with biological and pharmacological properties, such as antioxidant, anti-inflammatory, antimicrobial, antimalarial, and anticarcinogenic properties [24]. Due to its hydrophobic nature, curcumin has very low solubility in water and its chemical stability has been reported to be affected by external factors such as pH, exposure to light, temperature and oxygen [25,26]. Curcumin has poor bioavailability due to inefficient absorption at the intestinal track and for that reason it is commonly administered with digestible lipids that facilitate the solubilization and transport of this phenolic compound to the epithelial tissue [25].

Electrospinning processing is suitable for the production of continuous and functional nano-microfibers, from a wide range of (bio)polymers [27] and small molecules such as phospholipids [10,11,12]. The fabrication of electrospun phospholipid fibers has been initially reported using DMF:CHCl_3_ solvents [11]. Recently, it was demonstrated that the morphological properties of electropsun phospholipid fibers could be controlled using solvents with different polarities such as isooctane, cyclohexane and limonene and by the application of a co-axial solvent electrospinning [11]. The mechanical properties of phospholipid microfibers were investigated by nanoindentation using Atomic Force Microscopy [12]. It was found that these fibers have an elastic modulus of 17.26 MPa and were stable in ambient conditions, preserving the modulus of elasticity up to 24 h [12]. In another study, Yu et al. [28] mixed polyvinylpyrrolidone (PVP) with soybean lecithin to create a fibrous network by electrospinning. Formation of liposomes and vesicles with a very narrow distribution between 120–370 nm was observed after immersion of lecithin/PVP fibers in water. To increase the stability of electrospun lipid based systems, Zhang et al. applied a hybridization strategy to produce electrospun cholesteryl-succinyl silane (CSS) nanofibers [29].

Electrospun fibers have been used for encapsulation and controlled release of bioactives [27]. The encapsulation of curcumin within electrospun fibers using Chitosan/Phospholipids [13], polylactic acid (PLA) [30,31], polyvinyl pyrrolidone [32], blends of amaranth protein isolate/pullulan [33], and cellulose acetate [34], as well as the encapsulation of the vanillin/cyclodextrin inclusion complex (vanillin/CD-IC) within electrospun polyvinyl alcohol (PVA) [35] fibers has been reported.

This study aimed to develop electrospun phospholipid microfibers to encapsulate vanillin and curcumin as model phenolic compounds and investigate their morphology, release and antioxidant properties.

## 2. Results and Discussion

### 2.1. Morphology

**Figure 1:**
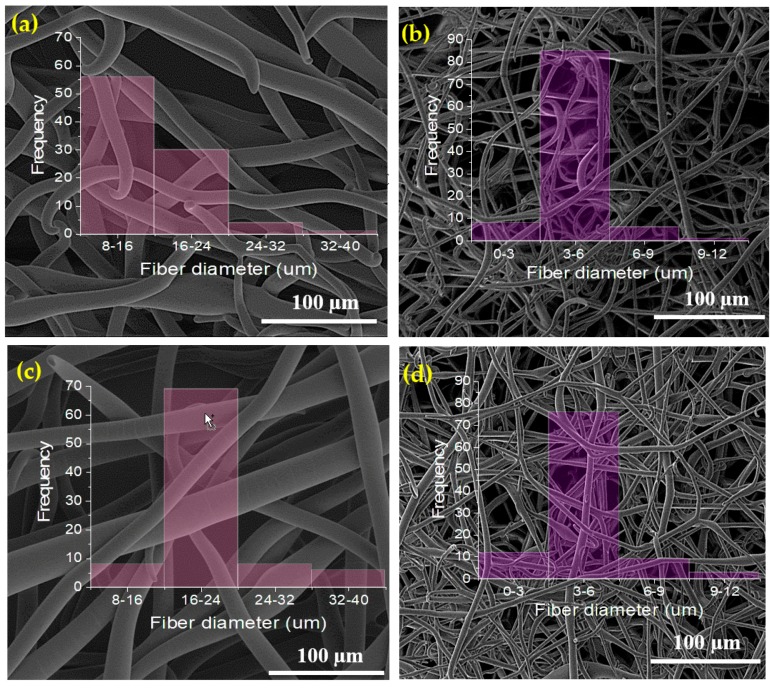
SEM images and corresponding histograms of the diameter distribution of electrospun phospholipid fibers prepared using limonene (**a**) and isooctane (**b**) as solvents encapsulating vanillin (**c**) and curcumin (**d**).

Phospholipid microfibers were obtained by electrospinning using limonene and isooctane solvents, as reported previously [11,12]. The fibrous structure (Figure 1a,b) is composed of individual, uniform and randomly oriented fibers with average diameters of 15.74 ± 4.68 µm and 4.51 ± 1.27 µm for limonene and isooctane, respectively.

The higher average diameter of electrospun asolectin phospholipid using limonene as solvent in comparison with isooctane is related to the dielectric constant (ɛ solvent) and the evaporation point (Bp solvent) of the solvents. Limonene has a higher evaporation point and dielectric constant (176 °C; 2.3) compared to isooctane (99 °C, 1.92), thus slower evaporation takes place resulting in fibers with higher average diameter as discussed in our previous studies [11].

Electrospun phospholipid fibers loaded with vanillin and curcumin were also developed with average diameters of 20.36 ± 5.4.5 and 4.42 ± 1.71 µm, respectively (Figure 1c,d). The inclusion of vanillin and curcumin on phospholipid solutions did not significantly change the morphology of electrospun phospholipid fibers, suggesting that the bioactives were efficiently encapsulated and well distributed within the microfibers. The increase in fiber diameter after encapsulation of bioactives within electropsun fibers has been well reported [13,36].

### 2.2. FTIR Analysis

**Figure 2:**
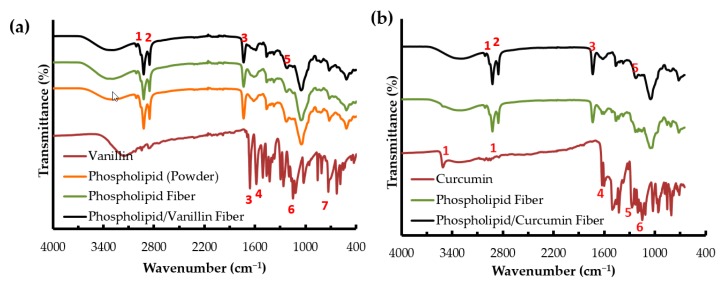
FTIR spectra of vanillin powder, asolectin powder, electrospun asolectin fiber and electrospun asolectin/vanillin fiber prepared using limonene (**a**) and curcumin powder, electrospun asolectin fiber and electrospun asolectin/curcumin fiber prepared using isooctane (**b**) as solvents. The red numbers in the figure are the assigned peaks listed in Table 1.

Figure 2a,b shows the FTIR spectra of asolectin, vanillin, curcumin, asolectin electrospun fibers made with isooctane and limonene (controls) and asolectin fibers with encapsulated vanillin and curcumin, respectively. Table 1 lists the assigned peaks.

The FTIR spectrum of pure asolectin powder showed the peaks at 3000 and 2800 cm^−1^ corresponding to the C-H stretching of CH_2_ groups, and the peaks at 1730 cm^−1^ and 1240 cm^−1^ corresponded to C=O stretching and PO_2_^−^ groups, respectively (Figure 2a) [37]. The features for asolectin fibers remained the same as the pure asolectin powder before the electrospinning process in both solvents, suggesting that the electrospinning process and the solvents did not change the physico-chemical properties of asolectin.

The FTIR spectrum of pure vanillin powder indicates characteristic peaks at 731, 1510 and 1590 cm^−1^ which correspond to the stretching vibration absorption of the benzene ring. The peak at 1660 cm^−1^ is attributed to the stretching vibration of C=O of the aldehyde group [38]. Also, the peak at 1150 cm^−1^ shows the presence of ether groups in pure vanillin (Figure 2a) [23,39].

For curcumin, the bands observed at 3085–3552 cm^−1^, 1601 cm^−1^, 1273 cm^−1^, and 1152 cm^−1^ are respectively attributed to the phenolic O-H stretching, stretching vibrations of the benzene ring, aromatic C-O stretching and C-O-C stretching modes (Figure 2b) [24] .

The FTIR spectra of electrospun asolectin fibers loaded with both vanillin and curcumin showed the same main peaks; therefore, it is assumed that both phenolic compounds were efficiently loaded within asolectin fibers and no interactions between the bioactives and the matrix occurred.

### 2.3. Encapsulation Efficiency (EE)

The encapsulation efficiency of vanillin and curcumin within asolectin microfibers was found to be of 85.23 ± 1.19% and 96.39 ± 2.81%, respectively. The relatively high encapsulation efficiency is related to the high solubility of vanillin and curcumin in limonene and isooctane, respectively. Consequently, the boactives could be efficiently dispersed within the fibers and well encapsulated.

The encapsulation efficiency of vanillin using electrospun almond gum/polyvinyl alcohol (PVA) composite nanofibers was reported to range from 68% to 75% for vanillin concentrations of 1% to 3% (*w*/*w*) respectively [40]. The EE of vanillin loaded within microcapsules of spray dried soy protein isolate/maltodextrin was 58.3% [41].

The EE of curcumin encapsulated within cellulose acetate electrospun fibers at concentrations of 5, 10, 15, and 20% *w*/*v* was reported to be of 101.9 ± 0.8%, 95.6 ± 2.5%, 91.4 ± 0.4%, and 90.8 ± 0.4%, respectively [34]. In another study, the EE of curcumin within liposomes was determined to range from 80.77 ± 4.12% to 82.32 ± 3.91% [42].

### 2.4. Total Antioxidant Capacity (TAC) Assay

**Figure 3:**
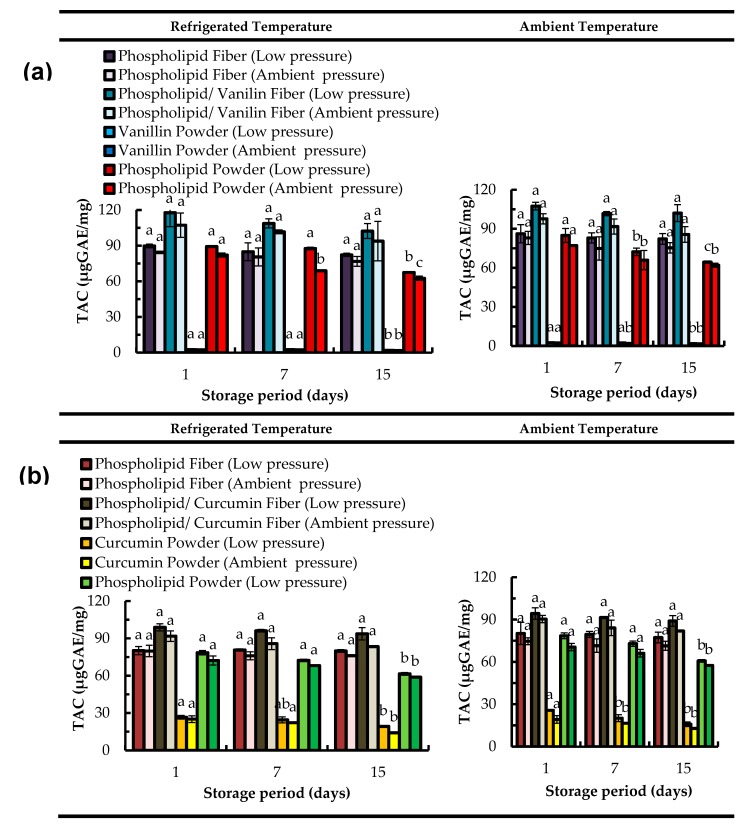
Total antioxidant capacity (TAC) over time of electropsun phospholipid fibers prepared using limonene (top graphs (**a**)) and isooctane (bottom graphs (**b**)) as solvent; Data are represented as mean ± SD [N = 3]; a–c: significant difference at *p* ≤ 0.05 in terms of total antioxidant capacity of each storage condition during storage time.

Figure 3 presents the effect of storage conditions (time, temperature and pressure) on the total antioxidant capacity of the both asolectin fibers with and without phenolic compounds. TAC was measured through the formation of the phosphomolybdenum complex and the reduction of Mo (VI) to Mo (V) by the antioxidant components in the phospholipid and phospholipid/bioactive specimens [34]. Several methods are available to measure the antioxidant capacity of food and biological systems [43,44]. The phosphomolybdenum method is used for extensive screening of the total antioxidant capacity of samples of very different origins and composition (hydrophobic and hydrophilic) from natural sources [45]. This is a simple low-cost method [46,47] and has been utilized for the determination of the antioxidant capacity of various compounds such as vitamin E [45], quercetin [29], curcumin [48], and flavonoid fractions of *Pistacia atlantica* fruit [43].

Electrospun asolectin fibers produced using limonene exhibited antioxidant capacity ranging from 76 to 89 µg Galic Acid Equivalent/mg of microfibers (µgGAE/mg) for samples stored at 4 °C, and from 75 to 86 µgGAE/mg when stored at ambient temperature (Figure 3). Similarly, the TAC determined for asolectin fibers prepared using isooctane ranged from 76 to 80 µgGAE/mg (4 °C) and from 71 to 80 µgGAE/mg (ambient temperature). These data suggested that the solvent does not play a significant role in TAC of phospholipid fibers. It is noteworthy that the slight differences of the TAC values of the (control) asolectin fibers prepared using limonene or isooctane as solvents, are related to the differences of the molecular mass of the solvents that has to be considered when using this analytical method. Phospholipids are known for their antioxidant properties [18,19,49] and asolectin that contains lecithin, cephalin and other phospholipids display antioxidant activity, as demonstrated previously [16,17].

For pure vanillin and curcumin powders (non-encapsulated), a decrease in TAC was observed over 15 days of storage. The TAC of vanillin stored at 4 °C was decreased by 23 (low pressure) and 25% (ambient pressure) from day 1 to day 15. At ambient temperature, the TAC of vanillin stored at low pressure and ambient pressure decreased by 30% and 26% respectively from 1 to day 15. After 15 days, curcumin stored at 4 °C was observed to significantly decrease its TAC to about 28% and 44% when stored at low pressure and ambient pressure, respectively. At room temperature non-encapsulated curcumin lost around 38% (at low pressure) and 33% (at room pressure) of its total antioxidant capacity from day 1 to day 15. Curcumin is a bioactive susceptible to oxidation [16] and its stability is known to be negatively affected by oxygen exposure [25,26].

However, after encapsulation of phenolic compounds, the TAC of asolectin fibers loaded with both curcumin and vanillin was found to be constant over time (Figure 3), suggesting that the antioxidant stability of the compounds can be maintained. Moreover, an increase in TAC was observed for fibers loaded with curcumin and vanillin, suggesting an improvement of antioxidant activity due to the combination of phospholipids with phenolic compounds. This is in accordance with previous studies where an improvement of TAC was confirmed by synergistic interactions of lecithins with other phenolic compounds such as tocopherols [17,50].

Previous studies showed that encapsulated curcumin could retain higher antioxidant stability in lecithin (antioxidant) stabilized emulsions compared to curcumin in Tween 20 (non-antioxidant) stabilized emulsions. The higher antioxidant activity of emulsifier could significantly lower the rate of radical permeation and consequently reduce the rate of oxidation of the encapsulated curcumin [16]. Figure 3 shows the TAC of curcumin/asolectin and curcumin/vanillin fibers over 15 days. The TAC assay was also conducted for 45 days and no evidence of reduction of TAC was observed (data not shown), suggesting that the phospholipids fibers and phospholipids/phenolic compounds fibers preserve their antioxidant activity for extended periods of time.

Antioxidant activity of phenolic compounds is known to be affected by the presence of oxygen and temperature. Figure 3 shows that no significant differences were observed for the TAC when microfibers-bioactives were stored at different temperatures (refrigerated, ambient) and pressures (vacuum, ambient pressure). However, the most favorable storage conditions for both curcumin and vanillin loaded in electrospun asolectin fibers were refrigerated temperature and low pressure, as slightly higher TAC values were determined.

**Figure 4:**
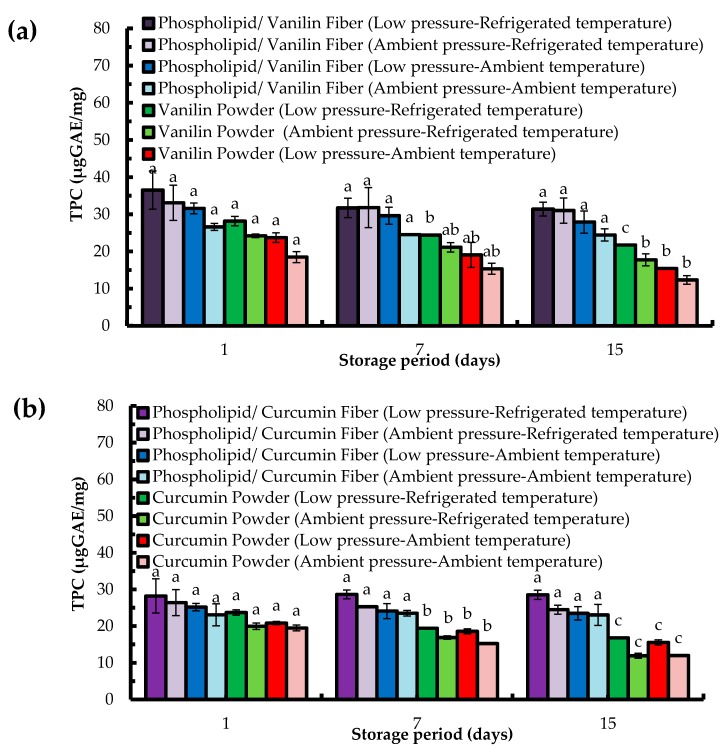
Total phenolic content (TPC) over time of electropsun phospholipid fibers prepared using limonene (**a**) and isooctan (**b**) as solvents; Data are represented as mean ± SD [N = 3]; a–c: significant difference at *p* ≤ 0.05 in terms of total phenolic content of each storage condition during storage time.

The total phenolic content (TPC) within the fibers over time was also determined based on the electron transfer from phospholipid and phospholipid/bioactive specimens to the complexed Mo (IV) present in the Folin–Ciocalteu reagent [51,52] (Figure 4). Similar to TAC (Figure 3), the total phenolic content was not changed significantly over time for the phenolics encapsulated within electropsun phospholipid fibers. However, for the non-encapsulated curcumin, a reduction of TPC by 40% from day 1 to day 15 was determined when this bioactive was stored in ambient pressure and refrigerated temperature (11.9 µgGAE/mg). The encapsulated curcumin displayed a TPC of 24.5 µgGAE/mg for the same storage conditions.

The TPC of non-encapsulated vanillin was observed to decrease by about 35% when this compound was stored at reduced pressure and room temperature, reaching the values of 15 µgGAE/mg after 15 days. The encapsulated vanillin stored at reduced pressure and room temperature exhibited a TPC of 28 µgGAE/mg (Figure 4).

Hence, TAC and TPC data demonstrate the potential of asolectin fibers to be used as antioxidant systems as well as efficient matrices for the encapsulation of phenolic compounds. Cases of preservation of TPC within electropsun fibers have been reported [53,54,55].

### 2.5. Stability of Phenolic Compounds Test under Storage by ^1^H-NMR

**Figure 5:**
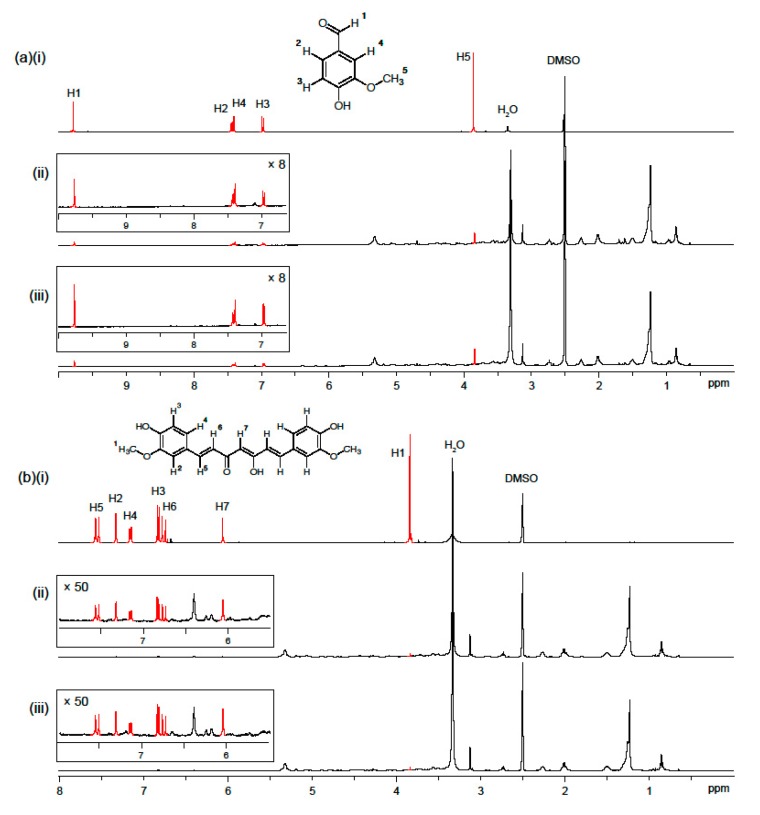
^1^H-NMR spectra (298 K, 400 MHz) in DMSO-*d*_6_ of vanillin (**a**(i)) and curcumin (**b**(i)); and electrospun phospholipid fibers with encapsulated vanillin and curcumin analyzed at different storage times: (ii) 1 day and (iii) 30 days.

The composition of the electrospun asolectin fibers with encapsulated curcumin or vanillin was analyzed using ^1^H-NMR spectroscopy. The chemical stability of vanillin and curcumin and the retention of these encapsulated phenolic compounds in the fibers upon storage were monitored. ^1^H-NMR spectra of the fibers in DMSO-*d*_6_ solution were obtained after increasing storage times at room temperature ranging from 1 day to 30 days (Figure 5). Figure 5a(i),b(i) shows the assigned ^1^H-NMR spectra of vanillin and curcumin as references. Figure 5a(ii),b(ii) shows the spectra for each fiber after 1 day of storage. In the region 0.5–3 ppm, several peaks corresponding to the aliphatic tails of the phospholipids can be seen. Highlighted in red are the peaks corresponding to the phenolic compounds. After 30 days of storage (Figure 5a(iii),b(iii)), the spectra have not altered. This suggests that not only are the encapsulated curcumin and vanillin chemically stable over the 30 days storage time, but the concentration of encapsulated phenolic compounds does not decrease under the storage conditions. This finding is in agreement with the total phenolic content data (TPC) measured at room temperature and pressure (Figure 3), suggesting that asolectin fibers were effective matrices to prolong the stability of vanillin and curcumin.

### 2.6. In Vitro Release Study

**Figure 6:**
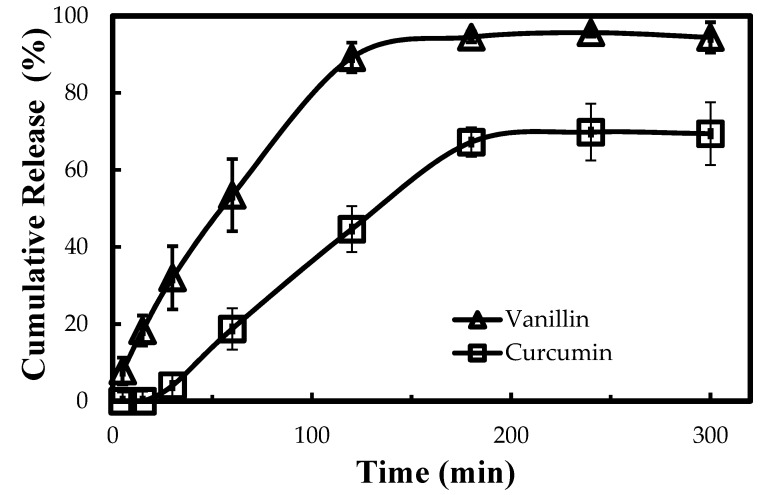
Cumulative release of vanillin and curcumin from phospholipid fibers into phosphate buffered saline (PBS), pH = 7.6 (n = 3). The error bars in the figure represent the standard deviation (SD).

The release profiles at 37 °C of vanillin and curcumin from asolectin microfibers are shown in Figure 6. For both vanillin and curcumin, a steady increase in the release of the bioactives was observed up to 240 min. At this time, the released amount of vanillin was 95% while the released amount of curcumin was 70%. From 240 to 300 min, only a slight increase of the released bioactives was observed.

The lower percentage of released curcumin compared to vanillin could be attributed to its hydrophobic nature. Similar curcumin release profiles showing a steady increase of curcumin released from electrospun nanofibers, produced using chitosan/phospholipid [13], blends of amaranth protein isolate (API)/pullulan [33] and PLGA copolymer solutions [56] have been reported. On the other hand, a higher percentage of release (near 100%) of vanillin from almond gum/polyvinyl alcohol (PVA) electrospun fibers has been observed in aqueous media, due to the higher solubility and higher swelling degree of almond gum/PVA/vanillin nanofiber in distilled water [40].

**Figure 7:**
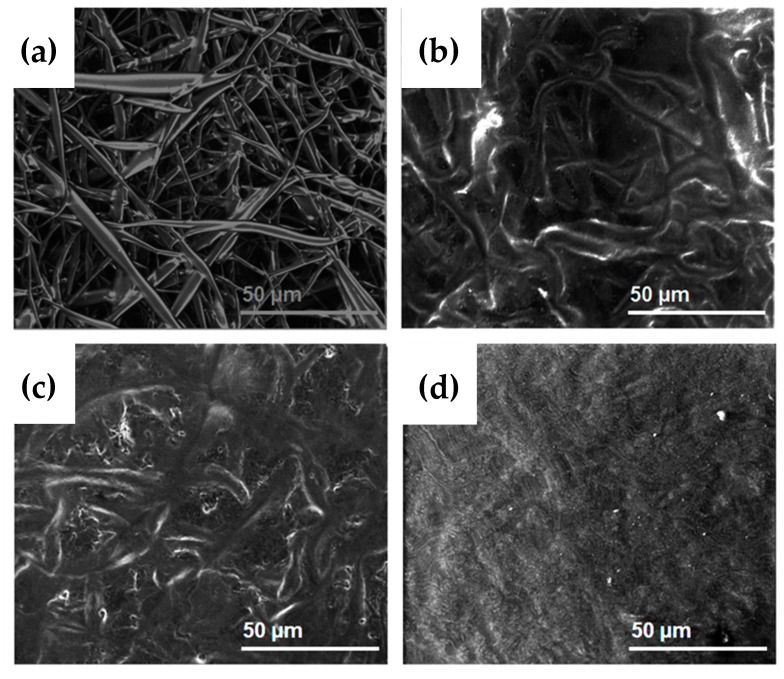
Environmental SEM (ESEM) images of elestrospun asolectin microfiber fibers (**a**), showing its morphology after immersion in PBS for 1 h (**b**), 2 h (**c**) and 4 h (**d**).

The release mechanism of the phenolic compounds from electrospun asolectin fibers was analyzed using the Korsmeyer–Peppas model [57]. This model concerns the release of drugs from cylindrical structures and predicts whether the release of the compound from a matrix follows Fickian diffusion, through determination of the coefficient “n” estimated from linear regression of the log(Cumulative Release) as a function of log(Time). The “n” determined from both release curves was above 0.45, with a correlation value (*R*^2^) of 0.98 and 0.99 for vanillin and curcumin respectively, suggesting that the release mechanism of both bioactives was mainly due to the swelling of the phospholipid fibers that influenced the release of bioactives. Wongsasulak and co-authors [58] also reported that the swelling of the matrix (electrospun polymers of zein, poly(ethylene oxide), and chitosan) triggered the release of the bioactive (α-tocopherol) according to Korsmeyer–Peppas model. To confirm the changes in morphology of phospholipid fibers after immersion in PBS, Environmental SEM (ESEM) photographs were taken at different time points of immersion (Figure 7). After one hour in PBS, fibers were observed to slightly increase their diameter as a result of the swelling of the matrix. At the end of 2 h, the progressive water absorption was observed (Figure 7c) resulting in the loss of fibril-like shape, that was further observed at the end of 4 hours of immersion in PBS (Figure 7d). It is to be noted that a previous study suggested that electrospun phospholipid scaffolds, made from lethicin solutions, lack physical stability as a consequence of hydration and further breakdown of fibril-like structures in the presence of moisture [59].

Yu and co-authors reported the fabrication of electropsun composite fibers made of polyvinylpyrrolidone (PVP) and soybean lecithin [27] and observed the collapse of composite fibers after contact with water within less than one minute as a result of changes in the hydrophilic/hydrophobic nature. Our system was observed to last longer which allowed the sustained release of encapsulated bioactives for 360 min (Figure 6).

## 3. Materials and Methods

### 3.1. Materials

Asolectin from soybean (Sigma-Aldrich product nr: 11145, lot nr: BCB66221V) was used as received. It contains approximately lecithin (25–33%), cephalin and phosphatidylinositol, saturated fatty acids (24%), mono-unsaturated (14%) and poly-unsaturated fatty acids (62%). Methyl-4-(1-methylethenyl)-cyclohexene (limonene), isooctane, curcumin and vanillin were obtained from Sigma-Aldrich and used as received without further purification.

### 3.2. Preparation of Electrospinning Solutions

A total of 60% (*wt*/*wt*) asolectin phospholipid was dissolved in limonene and isooctane at room temperature and then, vanillin (3%, *wt*/*v*) or curcumin (0.5% *wt*/*v*) were added, respectively, and stirred for 15 min before electrospinning processing. Solutions of 60% (*wt*/*wt*) asolectin were also prepared in both limonene and isooctane without phenolic compounds. Isooctane and Limonene were selected as solvents due to their capability produce electrospun phospholipid fibers [11] and to dissolve curcumin and vanillin, respectively.

### 3.3. Electrospinning Processing

The electrospinning setup included a high voltage generator (ES50P-10W, Gamma High Voltage Research, Inc., Ormond Beach, FL, USA) to provide a voltage of 21 kV, and syringe pump (New Era Pump Systems, Inc., Farmingdale, NJ, USA) to feed the solutions at a flow rate of 0.01 mL/min. Phospholipids fibers were collected on a steel plate covered with aluminium foil placed at a distance of 10 cm from the end of the needle. A blunt end stainless steel needle (Proto Advantage, Ancaster, ON, Canada) with inner diameters between 0.8 mm to 0.4 mm was used. The electrospinning process was carried out at ambient conditions.

### 3.4. Morphology

The morphology of the electrospun phospholipid, phospholipid/vanillin and phospholipid/curcumin fibers was investigated using a Quanta FEG 3D scanning electron microscope (SEM). Samples were attached on metal stubs with double-sided adhesive carbon tape and coated with 6 nm of gold for better conductivity using a sputter coater (Leica Coater ACE 200). The average fiber diameter was calculated using image J analysis software (National Institutes of Health, MD, USA) measured at 100 different points for each image. Changes in morphology of electrospun phospholipid fibers were further monitored after contact in PBS by Environmental SEM (ESEM). Samples were mounted on aluminium stubs and placed upside down immersed in PBS for 1, 2 and 4 h prior to the visualization in the Quanta FEG 3D SEM.

### 3.5. Fourier Transform Infrared (FTIR) Spectroscopy

FTIR spectra in the transmission mode were recorded using a Perkin 124 Elmer Spectrum 100 spectrometer based on a Universal Attenuated Total Reflectance sensor 125 (UATR-FTIR). The infrared peaks were identified using Spectrum™ 10 software using 1 %T peak threshold. Spectra were plotted as percentage transmittance (%T) against wavenumber (cm^−1^).

### 3.6. Encapsulation Efficiency

The encapsulation efficiency of the phenolic compounds (vanillin and curcumin) in asolectin fibers was determined by extracting the bioactives from the fibers using water and ethanol in a sonication bath for 30 min prior to centrifugation at 4500 rpm for 15 min. The concentration of bioactives in the supernatant was determined using a UV-Vis spectrophotometer (U-1500, Hitachi, Tokyo, Japan), where absorbance of vanillin and curcumin were measured at wavelengths of 280 nm and 425 nm, respectively. Standard curves for vanillin and curcumin were prepared with concentrations ranging from 0–100 μg/mL. Encapsulation efficiency was calculated using the following Equation (1):

(1) $$\%\;\mathrm{Encapsulation}\mathrm{Efficiency}=\;\frac{\mathrm{Phenolic}\;\mathrm{compounds}\;\left(\mathrm{encapsulated}\right)}{\mathrm{Phenolic}\;\mathrm{compounds}\;\left(\mathrm{total}\right)}\times 100$$

### 3.7. Total Antioxidant Capacity Assay (TAC)

Antioxidant capacities of phospholipid powder, vanillin powder, curcumin powder, phospholipid fibers, phospholipid/vanillin fibers and phospholipid/curcumin fibers were evaluated by the method of Jayaprakasha et al., 2006 [48]. Asolectin fibers (with and without phenolic compounds) were stored at different pressures (ambient and vacuum) and temperatures (refrigerated 4 °C and room temperature) and their TAC was evaluated after 1, 7 and 15 days storage. An amount of 300 µL of diluted extracted solution prepared in methanol was added to an Eppendorf tube containing 3 mL reagent solution (0.6 M sulfuric acid, 28 mM sodium phosphate and 4 mM ammonium molybdate). The Eppendorf tubes were capped and incubated in a water bath at 95 °C for 90 min. Then, samples were cooled to room temperature and the absorbance of each sample was measured at 695 nm against a blank (1 mL of reagent solution and the appropriate volume of the same solvent used for the sample). Gallic acid was used as the reference.

### 3.8. Total Phenolic Content (TPC)

The total phenolic content of the stored samples at different pressures (ambient and vacuum) and temperatures (refrigerated at 4 °C and room temperature) were determined via a modified Folin–Ciocalteu method [51] for 1, 7, 15 and 45 days of their storage. Briefly, 0.3 mL of diluted extract solution (5 mg/mL) was mixed with 0.6 mL of deionized water and 0.5 mL of Folin-Ciocalteu reagent in a test tube and then 1.5 mL of 20% sodium carbonate aqueous solution was added and the volume was made up to 10 mL with deionized water. The samples were incubated for 30 min at room temperature in darkness and then absorbance measured at 760 nm using and UV–Vis spectrophotometer (U-1500, Hitachi, Tokyo, Japan). The determination of the phenolic content was obtained by using gallic acid as a standard.

### 3.9. Stability of Phenolic Compounds Test under Storage by ^1^H-NMR Spectroscopy

Asolectin, asolectin/vanillin and asolectin/curcumin fibers were stored in ambient conditions at room temperature in the laboratory for 30 days. After 1, 15 and 30 days of storage, small samples of fibers were analyzed by ^1^H-NMR spectroscopy (400 MHz NMR spectrometer, Bruker, Billerica, MA, USA) at 298 K in order to compare the amount of the remaining phenolic compounds in the samples during storage time. Samples for ^1^H-NMR spectroscopic analysis were prepared in DMSO-*d*_6_ at a concentration of 10 mg/mL. The samples were alternatively sonicated and heated in closed vials with a heat gun in order to dissolve the material. ^1^H-NMR spectra of vanillin and curcumin were recorded as references.

### 3.10. In Vitro Release Study

The in vitro release of phenolic compounds from asolectin fibers was determined by UV–Vis spectroscopy [13]. Briefly, asolectin fibers (15 mg) were included in dialysis bags (SpectraLab with a MWCO 6–8 kDa) that were further sealed and placed into 20 mL phosphate buffer saline (pH~7.4) in a test tube in a shaking water bath at 37 °C for 360 min. The 2-mL samples were collected at each interval time point and replaced by fresh media (PBS, 2 mL). The amount of phenolic compounds released in the supernatant was determined afterwards using a UV–Vis spectrophotometer (U-1500, Hitachi, Tokyo, Japan) at optical wavelengths of 280 and 425 for vanillin and curcumin, respectively. A calibration curve of phenolic compounds in phosphate buffer saline (pH~7.4) was constructed with a concentration range from 0–100 μg/mL. The experiments were performed in triplicate and the results were reported as average values ± standard deviation.

The mechanism of release was investigated following the Korsmeyer–Peppas model:

(2) $$\frac{\mathrm{Mt}}{M\infty }={\mathrm{kt}}^{n}$$

where Mt/M∞ is the fraction of drug released at time t, k is the rate constant and n is the release exponent. If n ≤ 0.45, the release mechanism follows a Fickian diffusion and for 0.45 < n < 0.89 the drug release follows a non-Fickian diffusion (anomalous transport) [35].

### 3.11. Statistical Analysis

Presented results are an average of at least three independent experiments and are presented as mean ± standard deviation. The results were analyzed with one-way ANOVA using Fisher’s test in Minitab software version 16 (Minitab Inc., State College, PA, USA). The significant differences between samples were considered at a significance level of *p* ≤ 0.05.

## 4. Conclusions

In this study, electrospun phospholipid (asolectin) microfibers were investigated as encapsulation and antioxidant matrices for phenolic compounds, such as vanillin and curcumin. Asolectin fibers were observed to have antioxidant properties. Such antioxidant properties were improved after the encapsulation of the phenolic compounds, as observed from TAC and TPC assays. The antioxidant capacity of curcumin/phospholipid and vanillin/phospholipid microfibers was observed to remain stable over time at different temperatures (refrigerated, ambient) and pressures (vacuum, ambient), while the pristine non-encapsulated phenolic compounds decreased their TAC and TPC values. Moreover, the phospholipid matrix permitted the release of both curcumin and vanillin upon aqueous emersion, mainly due to the swelling of the phospholipid fibers that triggered the diffusion of bioactives. The above studies confirm the efficacy of electrospun phospholipid microfibers as encapsulation and antioxidant systems.

## Figures and Tables

**Table 1:** IR peak assignment of the electrospun phospholipid (Phos) fibers.

Peak Number	Group Frequency (cm^−1^)							Assignment
	Vanillin Powder	Curcumin Powder	Phos Powder	Phos (Limonene) Fiber	Phos (Isooctane) Fiber	Phos/Vanillin Fiber	Phos/CurcuminFiber	
1	-	3085–3552	3001	3000	3000	3000	3000	phenolic O-H stretching
2	-	-	2920 2850	2922 2852	2930 2859	2908 2847	2923 2853	C-H stretching of CH_2_ groups
3	1660	-	1730	1735	1746	1725	1740	C=O stretching of carbonyl groups
4	1590 1510	1601	-	-	-	-	-	stretching vibrations of the benzene ring
5		1273	1240	1230	1243	1205	1229	PO_2_^−^ groups; aromatic C-O stretching
6	1150	1152	-	-	-	-	-	C-O-C stretching
7	731	-	-	-	-	-	-	stretching vibrations of the benzene ring
